# Effects of positive end-expiratory pressure on gastric mucosal perfusion in acute respiratory distress syndrome

**DOI:** 10.1186/cc2905

**Published:** 2004-07-15

**Authors:** Alejandro Bruhn, Glenn Hernandez, Guillermo Bugedo, Luis Castillo

**Affiliations:** 1 Programa de Medicina Intensiva, Facultad de Medicina, Pontificia Universidad Católica de Chile, Santiago, Chile

**Keywords:** acute respiratory distress syndrome, gastric mucosal perfusion, positive end-expiratory pressure, tonometry

## Abstract

**Introduction:**

Positive end-expiratory pressure (PEEP) improves oxygenation and can prevent ventilator-induced lung injury in patients with acute respiratory distress syndrome (ARDS). Nevertheless, PEEP can also induce detrimental effects by its influence on the cardiovascular system. The purpose of this study was to assess the effects of PEEP on gastric mucosal perfusion while applying a protective ventilatory strategy in patients with ARDS.

**Methods:**

Eight patients were included. A pressure–volume curve was traced and ideal PEEP, defined as lower inflection point + 2 cmH_2_O, was determined. Gastric tonometry was measured continuously (Tonocap). After baseline measurements, 10, 15 and 20 cmH_2_O PEEP and ideal PEEP were applied for 30 min each. By the end of each period, hemodynamic, CO_2 _gap (gastric minus arterial partial pressures), and ventilatory measurements were performed.

**Results:**

PEEP had no effect on CO_2 _gap (median [range], baseline: 19 [2–30] mmHg; PEEP 10: 19 [0–40] mmHg; PEEP 15: 18 [0–39] mmHg; PEEP 20: 17 [4–39] mmHg; ideal PEEP: 19 [9–39] mmHg; *P *= 0.18). Cardiac index also remained unchanged (baseline: 4.6 [2.5–6.3] l min^-1 ^m^-2^; PEEP 10: 4.5 [2.5–6.9] l min^-1 ^m^-2^; PEEP 15: 4.3 [2–6.8] l min^-1 ^m^-2^; PEEP 20: 4.7 [2.4–6.2] l min^-1 ^m^-2^; ideal PEEP: 5.1 [2.1–6.3] l min^-1 ^m^-2^; *P *= 0.08). One patient did not complete the protocol because of hypotension.

**Conclusion:**

PEEP of 10–20 cmH_2_O does not affect gastric mucosal perfusion and is hemodynamically well tolerated in most patients with ARDS, including those receiving adrenergic drugs.

## Introduction

Recent studies have shown that lung protective strategies using low tidal volumes and high levels of positive end-expiratory pressure (PEEP) reduce mortality and are becoming standard practice in patients with acute respiratory distress syndrome (ARDS) [1,2].

Although PEEP improves arterial oxygenation, it can adversely affect systemic hemodynamics, reducing venous return and cardiac output. These effects are proportional to the PEEP level. Regional perfusion can also be affected by PEEP, independently of cardiac output changes. The splanchnic perfusion is particularly sensitive, and any reduction can compromise its barrier function, promote bacterial translocation, and contribute to the development of multiple organ failure [3]. In experimental models, PEEP has markedly decreased mesenteric and portal blood flow, despite only moderate reductions in cardiac output [4-8]. Similar results have been reported in patients without lung injury [9,10]. These effects are usually dose related, becoming more pronounced with PEEP levels around 20 cmH_2_O.

Kiefer reported that PEEP did not significantly alter splanchnic blood flow in six patients with acute lung injury [11]. Nevertheless, caution should be exercised in extending these results to clinical practice, because only hemodynamically stable patients without adrenergic drugs were studied, and PEEP levels never exceeded 14 cmH_2_O [12].

Our aim was to evaluate the effects of PEEP levels up to 20 cmH_2_O on gastric mucosal perfusion and systemic hemodynamics in mechanically ventilated patients with ARDS under hemodynamic support.

## Methods

### Patients

The study was approved by the Ethics Committee of the Medicine Faculty and was performed in the Surgical Intensive Care Unit of the Catholic University Hospital of Chile.

Adult mechanically ventilated patients were considered eligible for the study if they met the following criteria for ARDS during the 24 hours that preceded the study: acute onset of respiratory failure; diffuse bilateral infiltrates in the chest radiograph; a ratio of partial pressure of O_2 _(PaO_2_) to fraction of inspired oxygen (FiO_2_) of less than 200 mmHg; and a pulmonary arterial occlusion pressure less than 18 mmHg and no cardiac failure.

Hemodynamic monitoring included an arterial line and a pulmonary artery catheter (Baxter Edwards Critical-Care, Irvine, CA). Patients could be under vasopressor or inotropic support, but had to be hemodynamically stable for at least 3 hours before starting the protocol.

Patients were excluded if they had any of the following conditions: pregnancy, pre-existing respiratory dysfunction, cardiac index of less than 2.5 l min^-1 ^m^-2^, or were receiving enteral nutrition.

### Interventions

A nasogastric tonometer (TRIP^® ^Tonometry Catheter 14F, with biofilter connector for TONOCAP™ Monitor) was inserted into the stomach and connected to air automated tonometry (TONOCAP™ Monitor; Datex-Engstrom, Helsinki, Finland). All patients were sedated with midazolam and morphine, and paralyzed with rocuronium. Neuromuscular relaxation was measured by a TOF watch^® ^device. An intravenous 20 mg dose of famotidine was administered before starting the protocol. Patients were connected to volume-controlled mechanical ventilation (Servo 900 C; Siemens, Solna, Sweden). A pressure–volume curve was obtained for each patient by the airway occlusion technique [13], and ideal PEEP was defined as the lower inflection point + 2 cmH_2_O, or 12 cmH_2_O if no lower inflection point was found.

PEEP levels of 10, 15, 20 cmH_2_O, and ideal PEEP, with tidal volumes of 8 ml kg^-1^, were applied in four consecutive 30 min periods, respectively. Respiratory rate was modified to maintain end tidal CO_2 _within ± 10 mmHg of basal. All patients were receiving a constant infusion of 6% hetastarch before the beginning of the study (40–80 ml h^-1^). Cardiac output was optimized before and during the trial by determining the respiratory variation of systolic arterial pressure [14]. Whenever the variation was more than 10% a 100 ml bolus of 6% hetastarch was infused and the volume status was reassessed. No change in adrenergic support was allowed during the protocol. If hypotension (mean arterial pressure < 65 mmHg) persisted for more than 1 min, the protocol was stopped.

### Measurements

At baseline, and at the end of each period, hemodynamic, ventilatory and tonometric measurements were performed, and arterial blood samples withdrawn. Hemodynamic records included mean arterial pressure, heart rate, cardiac output, pulmonary artery occlusion pressure, central venous pressure and left ventricular stroke work index. Cardiac output was measured by thermodilution as the average of three values obtained after injections of 10 ml of 5% dextrose in water at room temperature. Mean airway pressure, oxygenation index and PEEP levels were registered. Oxygenation index was calculated as mean airway pressure × FiO_2 _× 100/PaO_2_. The CO_2 _gap (gastric partial pressure of CO_2 _[pCO_2_] minus arterial pCO_2_) was calculated by comparing simultaneous measurements of tonometric gastric mucosal pCO_2 _and arterial pCO_2_.

### Statistical analysis

Results are presented as median and range. The software Statview 5.0 was used to perform the statistical analysis. Nonparametric tests were used because of the small sample size. Data were analyzed with a Friedman test followed by a Wilcoxon signed-rank test if necessary. Results were considered statistically significant at *P *< 0.05.

## Results

Eight patients with ARDS were enrolled. They had a median (range) age of 63.5 years (23–86), and an Acute Physiology and Chronic Health Evaluation II score of 14 (7–20) at admission to the intensive care unit. On the day of the study they had a median Sepsis-related Organ Failure Assessment (SOFA) [15] score of 10 (7–13). All patients fulfilled criteria for ARDS, as defined by the inclusion criteria, during the 24 hours before the study and they had been on mechanical ventilation for 32 (12–72) hours. They were being ventilated with a median PEEP level of 9 (4–12) cmH_2_O, they had a PaO_2_/FiO_2 _ratio of 235 (144–388) mmHg and their respiratory system compliance was 45 (27–60) ml per cmH_2_O. Seven patients had sepsis (two pneumonia and five extrapulmonary sepsis), and one a severe thoracic trauma. Of the septic patients, six were in septic shock. Characteristics of individual patients are shown in Table 1.

**Table 1:** Baseline characteristics of the patients

Patient	Age (years)	Sex	Diagnosis	APACHE II	SOFA	PaO_2_/FiO_2 _(mmHg)	pH	Bicarbonate (mEq/L)	PEEP (cmH_2_O)	Crs (ml/cmH_2_O)	LIP (cmH_2_O)	Vasopressors/inotropes^a^	Outcome (S/NS)
1	55	M	Hepatic lobectomy	14	13	144	7.38	25.4	10	51	10	NA 0.08	S
												Dbt 3.3	
2	23	F	Peritonitis	20	10	388	7.36	23.5	8	32	10	NA 0.12	S
3	32	M	Mucormycosis and sepsis	7	7	282	7.42	21.5	6	60	6	NA 0.09	S
4	68	F	Acute pancreatitis	9	13	208	7.38	20.4	10	40	NL	NA 0.2	NS
5	59	F	Pneumonia and sepsis	16	8	197	7.28	25.5	10	55	NL	NA 0.03; Dp 6.8; Dbt 3.4	S
6	68	M	Thoracic trauma	14	10	289	7.36	21.6	4	37	13	NA 0.05	S
7	72	M	Sepsis	17	9	263	7.25	13.8	4	50	8	Dbt 5.4	S
8	86	M	Pneumonia and sepsis	14	12	150	7.37	20.3	12	27	13	NA 0.02	NS

APACHE, Acute Physiology and Chronic Health Evaluation; Crs, Respiratory system compliance; Dbt, dobutamine; Dp, dopamine; LIP, lower inflection point; NE, norepinephrine (noradrenaline); NL, no LIP found; NS, not significant; PEEP, positive end-expiratory pressure; S, significant; SOFA, Sepsis-related Organ Failure Assessment. ^a^Doses are in μg kg^-1 ^min^-1^.

**Figure 1:**
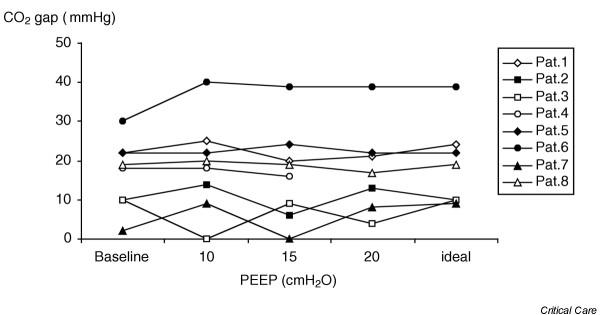
Individual changes in CO_2 _gap (gastric pCO_2 _minus arterial pCO_2_) with different positive end-expiratory pressure levels.

**Figure 2:**
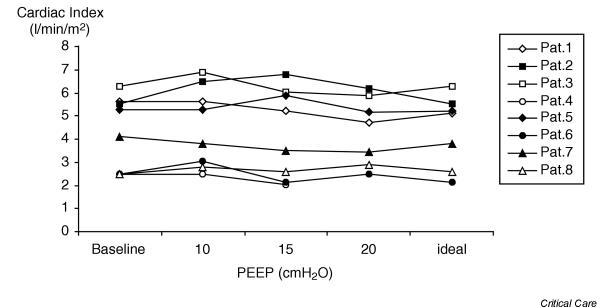
Individual changes in cardiac index with different positive end-expiratory pressure levels.

No changes in cardiac index or in CO_2 _gap were found at any of the study periods (Table 2). Oxygenation index, mean arterial pressure, pulmonary mean arterial pressure, pulmonary artery occlusion pressure, central venous pressure and left ventricular stroke work index also remained stable through the study. Only mean airway pressure and PaO_2_/FiO_2 _ratio differed between periods, as expected. Five patients required a 100 ml bolus of hetastarch during the trial; in no patient was it necessary to repeat it. Individual changes in CO_2 _gap and cardiac index are presented in Figs 1 and 2, respectively. At baseline three patients had already a CO_2 _gap of more than 20 mmHg. After starting the protocol with 10 cmH_2_O PEEP, patient 6, who was previously being ventilated with 4 cmH_2_O PEEP, had a further increase in CO_2 _gap. When PEEP was increased from 10 to 15 cmH_2_O, six patients decreased their CO_2 _gap and two increased it. When PEEP was increased from 15 to 20 cmH_2_O, three patients increased their CO_2 _gap, three decreased it and in one patient it remained unchanged. Patient 4 did not complete the protocol because of moderate hypotension (mean arterial pressure 60 mmHg) when PEEP was increased to 20 cmH_2_O. This patient recovered after an increased dose of norepinephrine (noradrenaline) and a return of PEEP to baseline levels.

**Table 2:** Respiratory, hemodynamic and tonometric measurements

Parameter	Baseline (*n *= 8)	PEEP 10 (*n *= 8)	PEEP 15 (*n *= 8)	PEEP 20 (*n *= 7)	Ideal PEEP (*n *= 7)	*P*
PEEP (cmH_2_O)	9 (4–12)	10	15	20	12 (8–15)	
Mean airway pressure (cmH_2_O)	13.2 (8–18.7)	14 (12–17)	19 (17–22.2)	24 (22–26.4)	16.2 (11.5–22.2)	0.0001^a^
OI (cmH_2_O per mmHg)	5.3 (2.9–12.4)	7 (3–14.5)	6.7 (4.1–12.3)	7 (5–12.3)	6.6 (2.9–12.3)	0.3
PaO_2_/FiO_2 _(mmHg)	235 (144–388)	210 (117–402)	285 (154–412)	333 (196–440)	243 (164–467)	0.0009^b^
PaCO_2 _(mmHg)	36 (31–54)	41 (28–63)	42 (31–66)	45 (32–60)	43 (28–52)	0.08
Cardiac index (l min^-1 ^m^-2^)	4.6 (2.5–6.3)	4.5 (2.5–6.9)	4.3 (2–6.8)	4.7 (2.4–6.2)	5.1 (2.1–6.3)	0.08
LVSWI (g m m^-2^)	45 (22–71)	43 (22–60)	40 (14–60)	36 (15–58)	42 (14–66)	0.13
MAP (mmHg)	79 (74–103)	81 (69–99)	74 (69–97)	74 (66–93)	73 (69–96)	0.24
PAOP (mmHg)	16 (10–19)	17 (8–22)	17 (11–23)	18 (12–26)	14 (11–23)	0.22
CVP (mmHg)	14 (9–17)	15 (7–19)	15 (9–24)	15 (10–19)	12 (8–18)	0.27
CO_2 _gap (mmHg)	19 (2–30)	19 (0–40)	18 (0–39)	17 (4–39)	19 (9–39)	0.18

Results are presented as median (range). CVP, central venous pressure; CO_2 _gap, arterial partial pressure of CO_2 _[pCO_2_] minus gastric pCO_2_; FiO_2_, fraction of inspired oxygen; LVSWI, left ventricular stroke work index; MAP, mean arterial pressure; OI, oxygenation index, defined as mean airway pressure × FiO_2 _× 100/arterial pCO_2_; PaO_2_, partial pressure of O_2_; PaCO_2_, partial pressure of CO_2_; PAOP, pulmonary arterial occlusion pressure; PEEP, positive end-expiratory pressure. ^a^*P *< 0.05 for all comparisons except baseline versus PEEP 10 and PEEP 10 versus ideal PEEP. ^b^*P *< 0.05 for all comparisons except baseline versus PEEP 10, baseline versus PEEP 15, baseline versus ideal PEEP, and PEEP 15 versus ideal PEEP.

Six of the eight patients studied survived (75%). The median length of stay in the intensive care unit was 17 (10–34) days and the median duration of mechanical ventilation was 9 (5–34) days.

## Discussion

Our results show that high PEEP levels (up to 20 cmH_2_O) do not compromise gastric mucosal perfusion, as assessed by tonometry, and do not affect systemic hemodynamics in most patients with ARDS. This is consistent with the findings of two other studies on the effects of PEEP on splanchnic perfusion in patients with ARDS. Nevertheless, in contrast with our study, neither of those studies included patients in septic shock or under adrenergic support [11,16].

Shock and cardiovascular dysfunction are frequently associated with ARDS. This is an important issue, because hemodynamic safety concerns could preclude the use of high or optimal PEEP levels in that setting, even if necessary. A major finding of our study is that PEEP levels up to 20 cmH_2_O can be well tolerated, even in patients with ARDS and septic shock. Nevertheless, our trial was relatively short and we cannot exclude the possibility that keeping high PEEP levels for a longer period might result in increased fluid requirements, which could be deleterious in the longer term.

Experimental and clinical research has demonstrated that in mechanically ventilated subjects without lung injury, PEEP decreases venous return and, secondarily, cardiac output [17-19]. In addition, Trager and colleagues showed that, in patients with acute respiratory failure associated with septic shock, high PEEP levels induced a decrease in cardiac output [20]. In contrast, we found no decrease in cardiac output in our patients tested with increasing PEEP levels when fluid administration was optimized according to respiratory variation in systolic arterial pressure. A similar result was reported by Kiefer and colleagues and by Akinci and colleagues [11,16]. Possible explanations for these contradictory results are a higher rate of fluid administration and the use of lower tidal volumes in the latter studies. Although we did not determine the upper inflection point of the pressure–volume curve, we think that by keeping tidal volume at 8 ml kg^-1 ^any overdistension of the lungs was minimized. Lung volumes are a critical component of the hemodynamic effects of ventilation [21]. Thus, it seems that it is possible to preserve cardiac output in patients with ARDS, despite the use of high PEEP levels, by optimizing fluid administration and limiting tidal volumes.

Gastric mucosal perfusion, as assessed by CO_2 _gap, also remained unchanged during the PEEP trial. This is consistent with the results reported by Kiefer and Akinci in similar studies. In all these studies cardiac output remained unchanged [11,16]. In contrast, Trager reported, in a series of septic shock patients with acute respiratory failure, that an increase in PEEP from 5 to 15 cmH_2_O induced a decrease in cardiac output associated to a decrease in hepatic vein O_2 _saturation and in hepatic glucose production [20]. It therefore seems that by avoiding decreases in cardiac output, splanchnic perfusion can be preserved in the majority of the patients.

In spite of the fact that no significant changes in CO_2 _gap or cardiac index were found during the protocol, when looking at the individual data certain patients evidenced an adverse effect when their PEEP level was increased. Patient 4, who had an extrapulmonary ARDS, presented hypotension when 20 cmH_2_O PEEP was applied. In this case, no simultaneous records of cardiac output or CO_2 _gap could be made for safety reasons (we immediately proceeded to decrease PEEP level). Patient 6, who had a pulmonary ARDS and who before starting the study had a 30 mmHg CO_2 _gap while being ventilated with 4 cmH_2_O PEEP, presented a further deterioration in CO_2 _gap after starting the protocol with 10 cmH_2_O PEEP, which was not associated with a decrease in cardiac output. Thereafter, the CO_2 _gap remained unchanged despite increasing PEEP up to 20 cmH_2_O. These events suggest that not all patients with ARDS can tolerate high PEEP levels; if required, careful hemodynamic monitoring including assessment of regional perfusion should be applied.

One major limitation of our study is the small number of patients studied. Thus, a type II error cannot be excluded. We did not perform any *a priori *power analysis because we had no estimation of the possible magnitude of the effects that PEEP could have on gastric tonometry.

Another limitation is the rather moderate severity of ARDS in our study. Although all patients fulfilled criteria for ARDS during the 24 hours that preceded the study, at inclusion their PaO_2_/FiO_2 _ratio and their respiratory system compliance were only moderately decreased. Two recent papers provide an explanation for this observation [22,23]. They show in patients diagnosed with ARDS that after a few hours of treatment with PEEP or a high FiO_2_, more than half of the patients present a PaO_2_/FiO_2 _ratio of more than 200 mmHg. In addition, respiratory system compliance increased by more than 10 ml per cmH_2_O after 6 hours of treatment with PEEP [23]. At inclusion our patients had already been ventilated with a median PEEP level of 9 cmH_2_O for more than 12 hours, which could have explained the rather improved respiratory performance at baseline. In any event, this improvement demonstrated a less severe ARDS. It is possible that more severely compromised patients might present a lower tolerance to high PEEP levels.

Other limitation is that tonometry was the sole method used to assess gastric mucosal perfusion. Nevertheless, Elizalde and colleagues showed that gastric mucosal blood flow, measured by laser Doppler flowmetry and by reflectance spectrophotometry, is well correlated with gastric intramucosal acidosis in mechanically ventilated patients [24].

## Conclusions

Our study supports the findings of previous studies suggesting that high PEEP levels do not affect splanchnic perfusion and are hemodynamically well tolerated in most patients with ARDS. Furthermore, our study shows that gastric mucosal perfusion can be well preserved while high PEEP levels are applied even in patients presenting cardiovascular dysfunction and receiving adrenergic support, which is a frequent occurrence in critical care. Nevertheless, two of the eight patients studied exhibited adverse effects during the PEEP trial, which highlights the importance of a close monitoring of systemic and regional perfusion while applying high PEEP levels to patients with ARDS. Future studies should assess the effects of PEEP on splanchnic perfusion in a longer term.

## Key messages

• High PEEP levels do not affect gastric mucosal perfusion and are hemodinamically well tolerated in most patients with ARDS

## Competing interests

None declared.

## Abbreviations

ARDS = acute respiratory distress syndrome; CO_2 _gap = gastric pCO_2 _minus arterial pCO_2_; FiO_2 _= fraction of inspired oxygen; PaO_2 _= partial pressure of O_2_; pCO_2 _= partial pressure of CO_2_; PEEP = positive end-expiratory pressure.
